# Prevalence and factors associated with asthma among adolescents and adults in Uganda: a general population based survey

**DOI:** 10.1186/s12889-019-6562-2

**Published:** 2019-02-22

**Authors:** Bruce J. Kirenga, Corina de Jong, Winceslaus Katagira, Samuel Kasozi, Levicatus Mugenyi, Marike Boezen, Thys van der Molen, Moses R. Kamya

**Affiliations:** 1 Makerere University Lung Institute & Division of Pulmonary Medicine, Department of Medicine, Makerere University College of Health Sciences, Kampala, Uganda; 2 GRIAC-Primary Care, department of General Practice and Elderly Care, University of Groningen, University Medical Center Groningen (UMCG), Groningen, The Netherlands; 3 Groningen Research Institute for Asthma and FIXED AIRFLOW OBSTRUCTION (GRIAC), University of Groningen, University Medical Center Groningen (UMCG), Groningen, The Netherlands; 4 Makerere University Lung Institute, Makerere University College of Health Sciences, Kampala, Uganda; 5 Makerere University Lung Institute, Makerere University College of Health Sciences, Kampala, Uganda; 6 Center for Statistics, Interuniversity Institute for Biostatistics and statistical Bioinformatics, UHasselt (Hasselt University), Diepenbeek, Belgium; 7 Department of Epidemiology, University of Groningen, Groningen, The Netherlands; 8 Groningen Research Institute for Asthma and COPD (GRIAC), University of Groningen, University Medical Center Groningen (UMCG), Groningen, The Netherlands

**Keywords:** Asthma, Prevalence, Uganda

## Abstract

**Background:**

Recent large-scale population data on the prevalence of asthma and its risk factors are lacking in Uganda. This survey was conducted to address this data gap.

**Methods:**

A general population based survey was conducted among people ≥12 years. A questionnaire was used to collect participants socio-demographics, respiratory symptoms, medical history, and known asthma risk factors. Participants who reported wheeze in the past 12 months, a physician diagnosis of asthma or current use of asthma medications were classified as having asthma. Asthmatics who were ≥ 35 years underwent spirometry to determine how many had fixed airflow obstruction (i.e. post bronchodilator forced expiratory volume in one second/forced vital capacity (FEV_1_/FVC) ratio < lower limit of normal (LLN). Descriptive statistics were used to summarize participants’ characteristics. Prevalence of asthma was calculated as a proportion of asthmatics over total survey population. To obtain factors independently associated with asthma, a random-effects model was fitted to the data.

**Results:**

Of the 3416 participants surveyed, 61.2% (2088) were female, median age was 30 years (IQR, 20–45) and 323 were found to have asthma. Sixteen people with asthma ≥35 years had fixed airflow obstruction. The prevalence of asthma was 11.0% (95% CI:8.9–13.2; males 10.3%, females 11.4%, urban 13.0% and rural 8.9%. Significantly more people with asthma smoked than non-asthmatics: 14.2% vs. 6.3%, *p* < 0.001, were exposed to biomass smoke: 28.0% vs. 20.0%, *p* < 0.001, had family history of asthma: 26.9% vs. 9.4%, *p*, < 0.001, had history of TB: 3.1% vs. 1.30%, *p* = 0.01, and had hypertension: 17.9% vs. 12.0%, *p* = 0. 003. In multivariate analysis smoking, (adjusted odds ratio (AOR), 3.26 (1.96–5.41, *p* < 0.001) family history of asthma, AOR 2.90 (98–4.22 *p*- < 0.001), nasal congestion, AOR 3.56 (2.51–5.06, *p* < 0.001), biomass smoke exposure, AOR 2.04 (1.29–3.21, *p* = 0.002) and urban residence, AOR 2.01(1.23–3.27, *p* = 0.005) were independently associated with asthma.

**Conclusion:**

Asthma is common in Uganda and is associated with smoking, biomass smoke exposure, urbanization, and allergic diseases. Health care systems should be strengthened to provide asthma care. Measures to reduce exposure to the identified associated factors are needed.

**Electronic supplementary material:**

The online version of this article (10.1186/s12889-019-6562-2) contains supplementary material, which is available to authorized users.

## Background

Asthma is estimated to affect 334 million people globally [1]. Recent large-scale population data on the prevalence of asthma and its risk factors are lacking in Uganda in particular and Africa in general. The world health survey conducted between 2002 and 2003 reported an asthma prevalence of 4–8% in the studied African countries [1]. A systematic review by Adeloye et al. found that the weighted mean prevalence of asthma was 7.0% in the rural areas (2.5–11.5) and 9.6% (3.9–15.2) in urban areas [2]. The same systematic review also indicates that the number of people suffering from asthma in Africa has increased from 74.4 million in 1990 to 119.3 million in 2010.

In addition to genetic susceptibility, several factors have been found to be associated with asthma [3]. These factors include exposure to allergens such as pollen and house dust mites, indoor air pollution (biomass smoke) and outdoor air pollution, tobacco smoking including second hand smoke (especially in children), urban residence and viral respiratory infections [3–7].

Diagnosing asthma is challenging as there is no gold standard test. A combination of characteristic clinical features and various tests (spirometry, airway inflammation, bronchial hyper-responsiveness testing, allergy testing) is used to arrive at a diagnosis in a clinical setting [8]. In surveys however, extensive clinical evaluation and testing is often not possible, hence surveys have relied mainly on symptom questionnaires. The three most commonly used questionnaires are those used in international study of asthma and allergy in childhood (ISAAC), the European community respiratory health survey (ECRHS) and the world health survey questionnaires [1, 9, 10].

To fill the data gap on asthma prevalence and its risk factors in Uganda, we aimed to conduct a national general population based survey.

## Methods

### Design and study participants

**Fig. 1:**
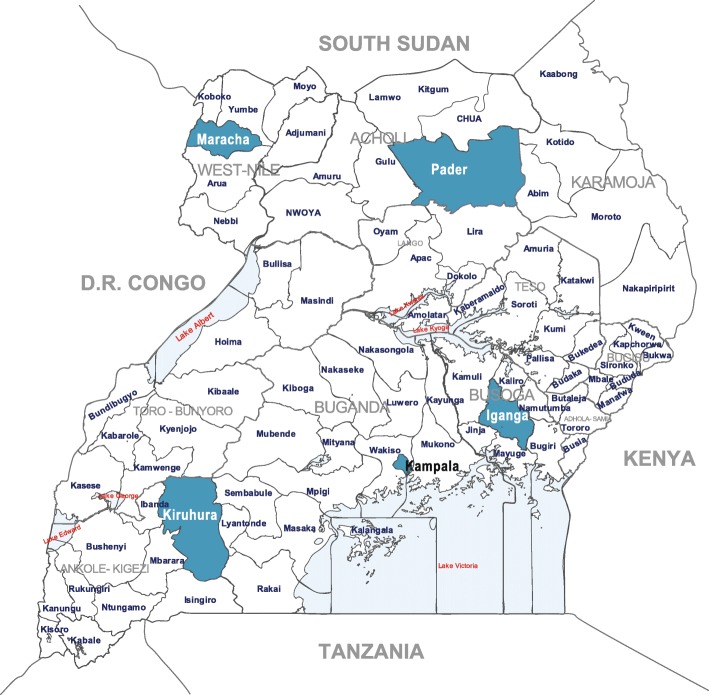
Survey districts (highlighted in blue), based on UN map of Uganda- including new districts by region

This study was a cross-sectional general population based survey in five districts in Uganda: Kampala (urban) and Iganga, Kiruhura, Maracha and Pader (rural), Fig. 1. The overall calculated sample size was 2936 participants (518 from each of 4 rural districts and 864 from Kampala) based on the assumption of an asthma prevalence of 8%, a precision of 0.03 and a design effect of 1.5 (to account for the cluster design). Clusters (villages) were selected by probability proportionate to size by Uganda Bureau of Statistics using the Uganda National population and housing census of 2014. Households within clusters were selected by simple random sampling from a household list generated by village leaders. All persons aged ≥12 who were members of selected household and provided written informed consent (and assent in case of minors) were surveyed. Exclusion criteria were: residency of congregation settings (schools, prisons, homes) and temporary residents (less than 2 weeks in household of selected villages). According to the Uganda National population and housing census of 2014, the average number of persons 12 years and older in a household was estimated to be 2.5 persons and the average number of households per cluster was 90 households. Based on these estimates we surveyed a total of 1408 households in 60 clusters across the country; 20 clusters in Kampala and 20 households from each of the clusters and in rural districts we surveyed 10 clusters and 25 households from each of the districts.

### Survey implementation

In this survey three field teams each comprising of one supervisor, two interviewers, one spirometry technician, one district tuberculosis and leprosy supervisor (DTLS), one local council 1 leader (LC1), one driver and community volunteers as needed was used. Each team surveyed one cluster per day (i.e. about 50 participants/day). The implementation of the survey commenced with the training of the survey teams. Thereafter, a pilot was undertaken to test survey human resources, study tools and the designed data system. After the pilot, adjustments to the tools and the data management system were made. The teams were retrained. Halfway into the survey, amid term review was conducted to inform the investigators of any needed adjustments and strategies to enhance the survey quality.

### Survey procedures

Sampled participants were interviewed by trained research assistants using a standardized questionnaire developed by adapting questions from internationally recognized questionnaires, namely the World Health Organization (WHO) health survey [1, 10], the ISAAC [10] and ECRHS surveys [9]. Participants who reported either wheeze in the last 12 months, history of current use of asthma medications at the time of the survey or history of ever having a physician diagnosis of asthma were considered to be asthmatics.

Anthropometric measurements were measured; height (measured without shoes to the nearest 0.1-cm using a stadiometer [SECA; Hamburg, Germany]) and weight (measured without shoes and in light clothing to the nearest 0.1 kg using a calibrated beam scale). Blood pressure (BP) was measured using an Omron automated sphygmomanometer model HEM-907, which has an adjustable cuff size. Participants assumed a resting seated posture ≥10 min prior to two sequential BP readings taken 10 min apart. We considered the average of the two BP readings as the individual’s BP. Participants with systolic BP > 130 and diastolic BP > 90 were considered to have hypertension for purposes of this analysis.

Participants who fulfilled the criteria for asthma on questionnaire and were ≥ 35 years underwent spirometry testing to assess for presence of fixed airflow obstruction. The 35 year cut off limit was chosen because fixed air flow obstruction increases with age and based on our previous surveys we found many persons with fixed airflow obstruction from age 35 years and older [11]. Participants identified as having asthma were referred to nearest health facilities for further evaluation and management. Spirometry was conducted and interpreted according to American Thoracic Society/European Respiratory Society guidelines using a Pneumotrac® spirometer with Spirotrac® V software (Vitalograph Ltd., Buckingham, United Kingdom) [12]. Spirometry was performed with participant seated and with a nose clip applied. Testing continued until at least three acceptable and reproducible blows with the largest and second-largest values for both forced vital capacity (FVC) and forced expiratory volume in 1 s (FEV _1)_ within 150 mL or no more than 5% difference; the largest values for FVC and FEV _1_ were considered the best and used for analysis. Spirometers were calibrated every morning with a 3 L syringe. Pre-bronchodilator spirometry was performed. Participants whose FEV_1_/FVC ratio was less than 80% underwent post bronchodilator spirometry (i.e. repeat spirometry 15 min after inhalation of 400 micrograms of inhaled salbutamol). On a daily basis, a physician reviewed all spirograms and those that did not meet the quality criteria were repeated the following day. Predicted parameters were based on NHANES III models as in built within the Spirotrac® V spirometers program used [13]. Participants whose post bronchodilator FEV_1_/FVC ratio was less than the LLN ie, participants below the fifth percentile of the predicted FEV _1_ /FVC ratio (calculated with GLI2012 Data Conversion software; version 3.3.1) were classified as having fixed airflow obstruction [14, 15]. However these participants were not excluded from asthma participants on this basis.

### Ethical approval

Ethics approval was obtained from the Mulago Hospital Research and Ethics committee and the Uganda National Council for Science and Technology. Participants provided written informed consent and were free to terminate study participation at any time during the study. For children between the ages of 12–18 years we obtained their written assent and written parental/legal guardian consent.

### Statistical analysis

The planned sample size was 2936 participants, sufficient to provide a precise national, rural vs. urban and male vs. female estimates assuming a national asthma prevalence of 8%. Urban setting was defined as any areas gazette by the government of Uganda as urban during the 2014 national housing and population census [16].

Prevalence of asthma was calculated as the proportion of participants with asthma in the survey population and presented with 95% confidence intervals (95% CI). Weighting to account for clustering due to the cluster design of the survey was performed. A weight, which is the reciprocal of the overall selection probability (p) was generated as 1/p where p = p_1_*p_2_*p_3_ with p_1,_ p_2_ and p_3_ being the probabilities of selecting a district, a cluster within a district, and a household within a cluster, respectively. Later, “*svy:*” command in Stata was used to apply the weights when estimating the prevalence and other statistics. Because weighted and unweighted prevalence estimates differed, we present the weighted prevalence estimates in this manuscript. Descriptive statistics was used to summarize participants’ characteristics.

To obtain factors independently associated with asthma, a random-effects model was fitted to the data [17]. All factors that were individually associated with asthma with *p*-value< 0.20 and demographic factors were subjected to multivariable analysis using a random-effects model. To arrive at a better fit, backward model building was conducted using likelihood ratio test (LRT), the multicollinearity was checked using the variance inflation factor (VIF). The results from a better fit and free from multicollinearity (VIF < 10) are presented as adjusted estimates. Data was analyzed using STATA (StataCorp. 2011. Stata Statistical Software: Release 12. College Station, TX: StataCorp LP).

## Results

### Characteristics of study participants

From September 15th to October 10th, 2016, 4310 participants were invited and 3416 participated (participation rate of 79.3%). Of 3416 participants, 61.2% (2088) were female, 22.78% (778) were of urban residence and the median age was 30 years (IQR 20–45). Further details of participants’ characteristics are shown in Table 1.

**Table 1:** Characteristics of study participants (social, demographic, risk factors, respiratory and allergy symptoms, and comorbidities)-Percent distribution by asthma status

Characteristic	Number	Percentage
Residence		
Urban	779	22.80
Rural	2637	77.20
Gender		
Male	1327	38.85
Female	2089	61.15
Age in years		
<15	372	10.89
15–24	883	25.86
25–34	681	19.94
35–44	577	16.90
45–54	475	13.91
55–64	225	5.92
65+	202	
Allergy symptoms		
Nasal congestion in the past 12 months	538	15.75
Itchy-watery eyes in the past 12 months	767	22.45
Skin rash in the past 12 months	408	11.96
Rash affected other areas	261	62.74
Respiratory symptoms		
Cough	711	20.83
Shortness of breath	309	9.05
Chest pain	873	25.56
Sputum production	257	7.52
Risk factors		
History of/passive smoking	242	7.09
Exposure to bio-mass†	698	20.44
Family history of asthmaΦ	377	11.05
History of TB treatment	50	1.45
HIV positive	103	3.02
Hypertensive	426	12.58

### Prevalence of asthma

**Fig. 2:**
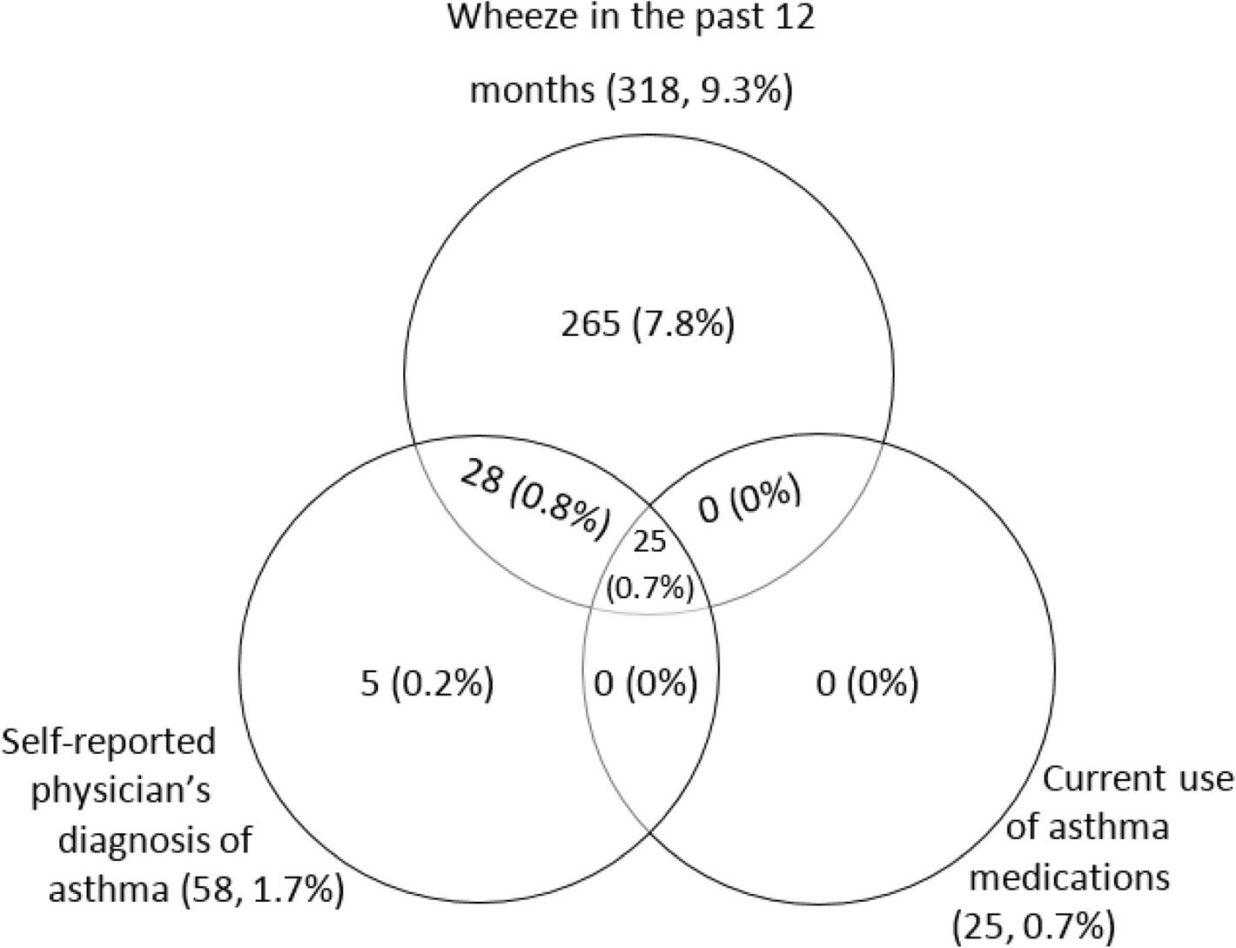
A Venn-diagram showing asthma prevalence by three diagnostic criteria and overlap between them

**Fig. 3:**
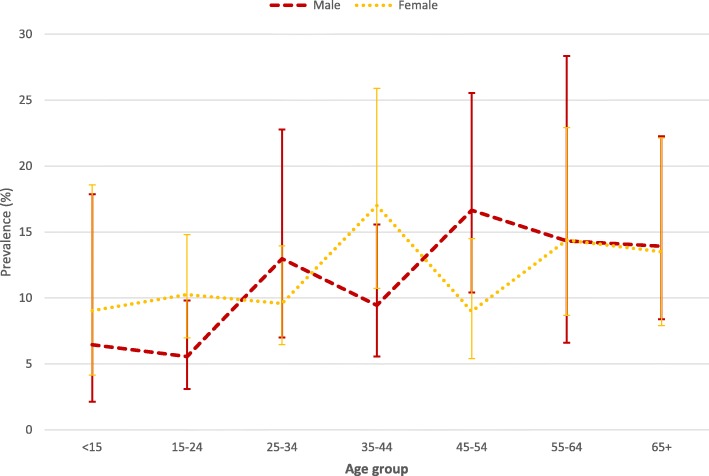
Prevalence of asthma by age group and gender

Overall 323 participants were found to have asthma. Three hundred and eighteen of 323 asthmatic participants (9.3%), 58/323 (1.7%), and 25/323 (0.7%) reported to have had wheezing the past 12 months, had ever had physician’s diagnosis of asthma, and were currently using asthma medications at the time of the survey, respectively. A Venn diagram showing overlaps between these three measures of asthma is presented in Fig. 2. The weighted prevalence of asthma was 11.02% (95% CI: 8.87–13.17), males 10.27% (95% CI: 7.88–12.65), females 11.40% (95% CI: 8.71–14.09), urban 12.99% (95% CI: 9.03–16.95), rural 8.86% (95% CI: 7.74–9.98), Table 2. Among both males and females, the asthma prevalence increased with increasing age, Fig. 3.

**Table 2:** Prevalence of asthma (Overall, by residence, gender, and age group

	Unweighted number	Weighted prevalence	
	n/N	%	95% CI
Overall	323/3416	11.02	8.87–13.17
Residence			
Rural	227/2637	8.86	7.74–9.98
Urban	96/779	12.99	9.03–16.95
Gender			
Male	114/1327	10.27	7.88–12.65
Female	209/2089	11.40	8.71–14.09
Age group			
<15	19/372	7.99	1.89–14.09
15–24	54/883	8.68	5.44–11.93
25–34	65/681	10.56	6.75–14.37
35–44	66/577	14.42	9.99–18.85
45–54	53/475	11.81	8.09–15.53
55–64	31/225	14.37	7.17–21.57
65+	35/201	13.66	8.06–19.25

### Comparison of characteristics of asthmatic and non-asthmatic survey participants

More asthmatics than non-asthmatics reported tobacco smoke exposure 14.2% vs. 6.3%, *p* < 0.001, biomass smoke exposure 28.0% vs. 19.7%, *p* < 0.001, family history of asthma 26.9% vs. 9.4%, *p*, <0.001, history of tuberculosis (TB) 3.1% vs. 1.3%, *p* = 0.010, and hypertension 17.9% vs. 12.0%, *p* = 0. 003, Additional file 1: Table S1.

The proportions of participants with allergy and respiratory symptoms by asthma status are presented in Additional file 1: Table S2A &2B. Nasal congestion in the past 12 months was reported by 40.3% of asthmatics vs. 13.2% non-asthmatics, *p* < 0.001). Itchy watery eyes were reported by 40.6% of asthmatics vs. 20.6% non-asthmatics, *p* < 0.001) while skin rash was reported by 20.7% of asthmatics vs. 11.0% non- asthmatics, *p* < 0.001. The proportions of the different respiratory symptoms by asthma vs. non-asthma status respectively were: cough (51.7% vs. 17.6%, *p* = < 0.001), shortness of breath (40.3% vs.5.8%, *p* < 0.001), chest pain (56.7% vs. 22.3%, *p* < 0.001) and sputum production (28.5% vs. 5.3%, *p* < 0.00).

### Factors associated with asthma

The factors independently associated with asthma in this survey as obtained from an adjusted random-effects model were: smoking, adjusted odds ratio (AOR) 3.26 (95% CI:1.96–5.41, *p* < 0.001), family history of asthma, AOR 2.90 (95% CI: 1.98–4.22 *p*- <0.001), nasal congestion in the past 12 months, AOR 3.56 (95% CI: 2.51–5.06, *p* < 0.001), biomass smoke exposure, AOR 2.04 (95% CI: 1.29–3.21, *p* = 0.02) and urban residence, AOR 2.01(95% CI: 1.23–3.27, *p* = 0.05), Table 3. All respiratory symptoms were associated with asthma, AORs (95% CIs) of: cough 2.41 (1.66–3.50, *p* < 0.001), shortness of breath 6.84 (4.57–10.23, *p* < 0.001), chest pain 3.00 (2.15–4.19, *p* < 0.001) and sputum production 1.81 (1.16–2.88, *p* = 0.009), Table 3. The factors associated with asthma in a model that considers only factors associated with asthma with a *p*-value less 0.05 at a bivariate stage are shown in Additional file 1: Table S3.

**Table 3:** Factors associated with asthma

Factors	With asthma	Without Asthma	Crude estimates		Adjusted estimates	
	n (%)	n (%)	Odds Ratio (95% CI)	*p*-value	Odds Ratio (95% CI)	*p*-value
History of /passive smoking						
Yes	46 (14.24)	196 (6.34)	2.80 (1.89–4.14)	<0.001	3.26 (1.96–5.41)	<0.001
No	277 (85.76)	2896 (93.66)	1		1	
Family history of asthmaΦ						
Yes	87 (26.93)	290 (9.39)	3.57 (2.68–4.76)	<0.001	2.90 (1.98–4.22)	<0.001
No	236 (73.07)	2800 (90.61)	1		1	
Nasal congestion in the past 12 months						
Yes	130 (40.25)	408 (13.20)	5.06 (3.79–6.75)	<0.001	3.56 (2.51–5.06)	<0.001
No	193 (59.75)	2684 (86.80)	1		1	
Cough						
Yes	167 (51.70)	544 (17.60)	6.48 (4.76–8.82)	<0.001	2.41 (1.66–3.50)	<0.001
No	156 (48.30)	2547 (82.40)	1		1	
Shortness of breath						
Yes	130 (40.25)	179 (5.79)	14.24 (9.90–20.50)		6.84 (4.57–10.23)	<0.001
No	193 (59.75)	2911 (94.21)	1		1	
Chest pain						
Yes	183 (56.66)	690 (22.32)	5.35 (4.04–7.08)	<0.001	3.00 (2.15–4.19)	<0.001
No	140 (43.34)	2402 (77.68)	1		1	
Sputum production						
Yes	92 (28.48)	165 (5.33)	9.01 (6.22–13.07)	<0.001	1.83 (1.16–2.89)	0.009
No	231 (71.52)	2928 (94.67)	1		1	
Exposure to bio-mass†						
Yes	90 (27.95)	608 (19.66)	1.60 (1.20–2.14)	0.001	2.04 (1.29–3.21)	0.002
No	232 (72.05)	2485 (80.34)	1		1	
Residence						
Urban	96 (29.72)	683 (22.08)	1.48 (1.11–1.97)	0.007	2.01 (1.23–3.27)	0.005
Rural	227 (70.28)	2410 (77.92)			1	
Sex:						
Female	209 (64.71)	1880 (60.78)	1.17 (0.91–1.50)	0.227	1.25 (0.89–1.74)	0.195
Male	114 (35.29)	1213 (39.22)	1		1	

### Fixed airflow obstruction

Of the 323 participants who were classified as having asthma on the questionnaire, 138 (42.72%) were 35 years and older and therefore eligible for spirometry. Of these, 120 (86.96%) underwent spirometry and 18(13.04%) did not. We obtained interpretable spirometry in 106 of the 120 (88.33%). After post bronchodilator testing, 16 of the 106 participants who underwent spirometry were confirmed to have fixed airflow obstruction (15.09%), 13(12.26%) had significantly reversible airflow obstruction (i.e. FEV_1_ reversibility of > 12% or > 200mls) and 9 (8.49%) had a restriction.

## Discussion

This survey found an asthma prevalence of 11.02% in Uganda, higher in urban areas than rural areas (12.99% vs. 8.86%) and among those aged 35–44 years (14.42%) compared to those either younger or older than those in this age group. No significant differences were found by gender (female 11.40% and male 10.27%). Significant associations were found between asthma and smoking, family history of asthma, nasal congestion, biomass smoke exposure, urban residence, and all respiratory symptoms. Asthmatic and non-asthmatic participants had statistically significant differences in the rates of history of TB (3.10% vs. 1.30% and hypertension (17.87% vs. 12.03%).

The prevalence of asthma and its higher rate in urban areas found in this survey are comparable to the prevalence reported in previous asthma surveys in Africa [1, 2, 18, 19]. There are no prior asthma surveys in Uganda among adolescents and adults apart from one report of history of asthma in pregnant women (6.0%. was reported) [20] Although the sex differences in asthma prevalence were small, the difference was bigger among rural participants (female 9.35% vs. 8.16% for males) than urban participants (females 13.22% vs. males 12.91%). The bigger difference in rural areas could be due to biomass smoke exposure, which is greater in females. Biomass smoke exposure has been found to be associated with asthma in this study and several previous studies [19, 21]. The smaller difference in urban areas could be attributed to higher ambient air pollution. We have previously shown that air quality in Kampala, where the urban sample was drawn, exceeds safety limits by 5 times [22].

Analysis of the relation between age and asthma shows that asthma peaked in the 35–44 age groups with another peak in those > 55 years. The peak in the 35–44 age group is previously reported [23]. The second peak of asthma that we observed in this study could be due to chronic obstructive pulmonary disease (COPD) that increases in prevalence with increasing age [24] and given the fact that we defined asthma by symptoms such as wheeze which can overlap with those of COPD. It is therefore possible that some of the patients that we counted as asthma could have had COPD. The prevalence of COPD has been found to be as high as 16% in some places in Uganda [11]. To address the issue of older asthmatics having COPD we analyzed the data taking all those who had fixed airflow obstruction as COPD and found that only 5% of all asthmatic could be reclassified as COPD. Our results therefore support other studies’ findings that asthma is an important respiratory disease in older people [25]. It must be noted however that fixed airflow obstruction can occur in asthmatics even in the absence of COPD due to airway remodeling with long standing asthma especially if care is suboptimal. There are several risk factors for this occurrence namely severe asthma, long-standing and poorly treated or untreated disease, late onset asthma, smoking, frequent exacerbations, ongoing exposures to asthma triggers, persistent eosinophilic airway inflammation and asthma-COPD overlap [19, 26–29]. In this survey 98.5% of the asthmatics were neither diagnosed nor on asthma treatment that could have led to fixed airflow obstruction.

This survey confirmed the association of several known risk factors with asthma namely smoking, biomass exposure, allergy, respiratory symptoms, and urban residence. We were also able to show a significant association between biomass smoke exposure and asthma. The rates of TB and hypertension were statistically significantly higher among asthmatics in comparison to non-asthmatics: TB (3.10% vs. 1.30%, *p* = 0.010) and hypertension (17.87% vs. 12.03%, p0.003). TB has been reported to be associated with asthma in previous studies including a large South Africa population based study [19, 30]. Although the data is limited, the association between hypertension and asthma has also been previously reported [31–33].

This survey had limitations of geographical coverage (only 5 districts included), not including questions to assess occupational asthma and being conducted in the wet season without comparison with the dry season. Although we had large numbers of males and females the overall proportion of males was lower in the sample. We adjusted for this difference in all analyses but this could have introduced a bias in the sex differences in the prevalence. Although, cross-sectional data cannot be used to draw conclusions on causality, the identified risk factors are well in line with previous prospective studies in other populations.

## Conclusion

Asthma is common in Uganda and is associated with smoking, biomass smoke exposure, urbanization, and allergic diseases. Health care systems should be strengthened to provide asthma care. Measures to reduce exposure to the identified associated factors are needed.

## Additional file

Additional file 1: This file contains 4 tables that present additional data obtained in the survey that we consider important to publish. Additional file 1: **Table S1** provides data of the comparison of the social, demographic and clinical characteristics of participants with asthma and those without asthma in the survey, Additional file 1: **Table S2**A presents allergy characteristics of the participants by asthma status while Additional file 1: **Table S2**B presents the respiratory symptoms of the participants by asthma status. Additional file 1: **Table S3** presents findings of a multivariate model of the factors associated with asthma considering only factors associated with asthma at the bivariate stage with *p*-value less than 0.05. (DOCX 38 kb)

## Abbreviations

AOR: Adjusted odds ratio
ATS: American Thoracic Society
CI: Confidence interval
COPD: Chronic obstructive pulmonary disease
ECRHS: European community respiratory health survey
ERS: European Respiratory Society
FEV_1_: Forced expiratory volume in the first second
FVC: Forced vital capacity
IQR: Interquartile range
ISAAC: International study of asthma and allergies in childhood
LRT: Likelihood ratio test
NHANES: National health and nutrition examination survey
TB: Tuberculosis
VIF: Variance inflation factor
WHO: World Health Organization
