# Supplementary material for: Prevalence and factors associated with asthma among adolescents and adults in Uganda: a general population based survey
Source: BMC Public Health. 2019 Feb 22;19:227. doi: 10.1186/s12889-019-6562-2 (PMC6387513; doi:10.1186/s12889-019-6562-2)
Supplement: Supplementary file 1 — This file contains 4 tables that present additional data obtained in the survey that we consider important to publish. Additional file 1: Table S1 provides data of the comparison of the social, demographic and clinical characteristics of participants with asthma and those without asthma in the survey, Additional file 1: Table S2A presents allergy characteristics of the participants by asthma status while Additional file 1: Table S2B presents the respiratory symptoms of the participants by asthma status. Additional file 1: Table S3 presents findings of a multivariate model of the factors associated with asthma considering only factors associated with asthma at the bivariate stage with p-value less than 0.05. (DOCX 38 kb) [file 12889_2019_6562_MOESM1_ESM.docx]

**Supplementary tables**

**Supplementary table 1. Comparison of social, demographic, risk factors, respiratory and allergy symptoms, and comorbidities characteristics of participants with and without asthma**

| **Characteristic** | **With asthma**  **n (%)** | **Without asthma**  **n (%)** | **p-value** |
| --- | --- | --- | --- |
| **Residence** |  |  |  |
| Urban | 96 (29.72) | 683 (22.08) | 0.002 |
| Rural | 227 (70.28) | 2410 (77.92) |  |
| **Gender** |  |  | 0.169 |
| Male | 114 (35.29) | 1213 (39.22) |  |
| Female | 209 (64.71) | 1880 (60.78) |  |
| **Age in years** |  |  |  |
| <15 | 19 (5.88) | 353 (11.42) | <0.001 |
| 15-24 | 54 (16.72) | 829 (26.81) |  |
| 25-34 | 65 (20.12) | 616 (19.92) |  |
| 35-44 | 66 (20.43) | 511 (16.53) |  |
| 45-54 | 53 (16.41) | 422 (13.65) |  |
| 55-64 | 31 (9.60) | 194 (6.27) |  |
| 65+ | 35 (10.84) | 167 (5.40) |  |
| **Allergy symptoms** |  |  |  |
| Nasal congestion in the past 12 months | 130 (40.25) | 408 (13.20) | <0.001 |
| Itchy-watery eyes in the past 12 months | 131 (40.56) | 636 (20.56) | <0.001 |
| Skin rash in the past 12 months | 67 (20.74) | 341 (11.04) | <0.001 |
| Rash affected other areas | 46 (65.71) | 215 (62.14) | 0.573 |
| **Respiratory symptoms** |  |  |  |
| Cough | 167 (51.70) | 544 (17.60) | <0.001 |
| Shortness of breath | 130 (40.25) | 179 (5.79) | <0.001 |
| Chest pain | 183 (56.66) | 690 (22.32) | <0.001 |
| Sputum production | 92 (28.48) | 165 (5.33) | <0.001 |
| **Risk factors** |  |  |  |
| History of /passive smoking | 46 (14.24) | 196 (6.34) | <0.001 |
| Exposure to bio-mass† | 90 (27.95) | 608 (19.66) | <0.001 |
| Family history of asthmaᶲ | 87 (26.93) | 290 (9.39) | <0.001 |
| History of TB treatment | 10 (3.10) | 40 (1.30) | 0.010 |
| HIV positive | 16 (4.95) | 87 (2.81) | 0.101 |
| Hypertensive | 57 (17.87) | 369 (12.03) | 0.003 |

**Supplementary table 2A. Allergy, Percent distribution of participants by region, gender and asthma status**

|  | | | | | | | | | |
| --- | --- | --- | --- | --- | --- | --- | --- | --- | --- |
| Allergies | | **With asthma** | | |  | **Without asthma** | | | P-value |
|  |  | Weighted percent | Unweighted percent | Unweighted number  n/N |  | Weighted percent | Unweighted percent | Unweighted number  n/N |  |
| **Allergy** |  |  |  |  |  |  |  |  |  |
| **Urban** |  |  |  |  |  |  |  |  |  |
| **Males** |  |  |  |  |  |  |  |  |  |
| Nasal congestion in the past 12 months | Yes | 52.23 | 53.85 | 14/26 |  | 18.34 | 18.23 | 33/181 | <0.001 |
|  | No | 47.77 | 46.15 | 12/26 |  | 81.66 | 81.77 | 148/181 |  |
| Itchy-watery eyes in the past 12 months | Yes | 39.10 | 38.46 | 10/26 |  | 16.27 | 16.02 | 29/181 | 0.006 |
|  | No | 60.90 | 61.54 | 16/26 |  | 83.73 | 83.98 | 152/181 |  |
| Skin rash in the past 12 months | Yes | 17.63 | 19.23 | 5/26 |  | 14.67 | 12.67 | 23/181 | 0.363 |
|  | No | 82.37 | 80.77 | 21/26 |  | 85.33 | 87.33 | 158/181 |  |
| Rash affected other areas | Yes | 58.20 | 60.00 | 3/5 |  | 47.50 | 50.00 | 12/24 | >0.999 |
|  | No | 41.80 | 40.00 | 2/5 |  | 52.50 | 50.00 | 12/24 |  |
| **Females** |  |  |  |  |  |  |  |  |  |
| Nasal congestion in the past 12 months | Yes | 60.80 | 60.00 | 42/70 |  | 20.94 | 21.12 | 106/502 | <0.001 |
|  | No | 39.20 | 40.00 | 28/70 |  | 79.06 | 78.88 | 396/502 |  |
| Itchy-watery eyes in the past 12 months | Yes | 48.39 | 47.14 | 33/70 |  | 21.11 | 21.31 | 107/502 | <0.001 |
|  | No | 51.61 | 52.86 | 37/70 |  | 78.89 | 78.69 | 395/502 |  |
| Skin rash in the past 12 months | Yes | 35.81 | 34.29 | 24/70 |  | 15.40 | 14.74 | 74/502 | <0.001 |
|  | No | 64.19 | 65.71 | 46/70 |  | 84.60 | 85.26 | 428/502 |  |
| Rash affected other areas | Yes | 64.46 | 64.00 | 16/25 |  | 57.88 | 58.11 | 43/74 | 0.604 |
|  | No | 35.54 | 36.00 | 9/25 |  | 42.12 | 41.89 | 31/74 |  |
| **Rural** |  |  |  |  |  |  |  |  |  |
| **Males** |  |  |  |  |  |  |  |  |  |
| Nasal congestion in the past 12 months | Yes | 28.59 | 29.55 | 26/88 |  | 10.26 | 11.34 | 117/1032 | <0.001 |
|  | No | 71.41 | 70.45 | 62/88 |  | 89.74 | 88.66 | 915/1032 |  |
| Itchy-watery eyes in the past 12 months | Yes | 34.16 | 35.23 | 31/88 |  | 19.46 | 20.74 | 214/1032 | 0.002 |
|  | No | 65.84 | 64.77 | 57/88 |  | 80.54 | 79.26 | 818/1032 |  |
| Skin rash in the past 12 months | Yes | 11.86 | 14.77 | 13/88 |  | 9.25 | 10.09 | 104/1031 | 0.168 |
|  | No | 88.14 | 85.23 | 75/88 |  | 90.75 | 89.91 | 927/1031 |  |
| Rash affected other areas | Yes | 83.42 | 83.33 | 10/12 |  | 62.65 | 64.76 | 68/105 | 0.332 |
|  | No | 16.58 | 16.67 | 2/12 |  | 37.35 | 35.24 | 37/105 |  |
| **Females** |  |  |  |  |  |  |  |  |  |
| Nasal congestion in the past 12 months | Yes | 38.10 | 34.53 | 48/139 |  | 9.81 | 11.04 | 152/1377 | <0.001 |
|  | No | 61.90 | 65.47 | 91/139 |  | 90.19 | 88.96 | 1225/1377 |  |
| Itchy-watery eyes in the past 12 months | Yes | 42.08 | 41.01 | 57/139 |  | 19.70 | 20.75 | 286/1378 | <0.001 |
|  | No | 57.92 | 58.99 | 82/139 |  | 80.30 | 79.25 | 1092/1378 |  |
| Skin rash in the past 12 months | Yes | 18.96 | 17.99 | 25/139 |  | 8.47 | 10.18 | 140/1375 | 0.005 |
|  | No | 81.04 | 82.01 | 114/139 |  | 91.53 | 89.82 | 1235/1375 |  |
| Rash affected other areas | Yes | 61.78 | 60.71 | 17/28 |  | 63.80 | 64.34 | 92/143 | 0.715 |
|  | No | 38.22 | 39.29 | 11/28 |  | 36.20 | 35.66 | 51/143 |  |

**Supplementary table 2B. Respiratory symptoms, Percent distribution of participants by region, gender and asthma status**

| **Respiratory symptoms** | | **With asthma** | | |  | **Without asthma** | | | P-value |
| --- | --- | --- | --- | --- | --- | --- | --- | --- | --- |
|  |  | Weighted percent | Unweighted percent | Unweighted number  n/N |  | Weighted percent | Unweighted percent | Unweighted number  n/N |  |
| **Urban** |  |  |  |  |  |  |  |  |  |
| **Males** |  |  |  |  |  |  |  |  |  |
| Cough | Yes | 39.52 | 42.31 | 11/26 |  | 11.34 | 11.11 | 20/180 | <0.001 |
|  | No | 60.48 | 57.69 | 15/26 |  | 88.66 | 88.89 | 160/180 |  |
| Shortness of breath | Yes | 30.16 | 30.77 | 8/26 |  | 5.00 | 4.44 | 8/180 | <0.001 |
|  | No | 69.84 | 69.23 | 18/26 |  | 95.00 | 95.56 | 172/180 |  |
| Chest pain | Yes | 47.35 | 46.15 | 12/26 |  | 12.62 | 12.15 | 22/181 | <0.001 |
|  | No | 52.65 | 53.85 | 14/26 |  | 87.38 | 87.85 | 159/181 |  |
| Sputum production | Yes | 25.91 | 26.92 | 7/26 |  | 5.81 | 5.52 | 10/181 | <0.001 |
|  | No | 74.09 | 73.08 | 19/26 |  | 94.19 | 94.48 | 171/181 |  |
| Wheezing | Yes | 48.82 | 50.00 | 13/26 |  | 0.00 | 0.00 | 0/181 | <0..001 |
|  | No | 51.18 | 50.00 | 13/26 |  | 100 | 100 | 181/181 |  |
| Hypertensive | Yes | 3.20 | 3.85 | 1/26 |  | 7.20 | 6.78 | 12/177 | >0.999 |
|  | No | 96.80 | 96.15 | 25/26 |  | 92.80 | 93.22 | 165/177 |  |
| **Females** |  |  |  |  |  |  |  |  |  |
| Cough | Yes | 33.93 | 32.86 | 23/70 |  | 13.92 | 13.94 | 70/502 | <0.001 |
|  | No | 66.07 | 67.14 | 47/70 |  | 86.08 | 86.06 | 432/502 |  |
| Shortness of breath | Yes | 40.87 | 41.43 | 29/70 |  | 5.71 | 5.79 | 29/501 | <0.001 |
|  | No | 59.13 | 58.57 | 41/70 |  | 94.29 | 94.21 | 472/501 |  |
| Chest pain | Yes | 42.22 | 41.43 | 29/70 |  | 14.38 | 13.94 | 70/502 | <0.001 |
|  | No | 57.78 | 58.57 | 41/70 |  | 85.62 | 86.06 | 432/502 |  |
| Sputum production | Yes | 16.66 | 15.71 | 11/70 |  | 5.45 | 5.18 | 26/502 | 0.001 |
|  | No | 83.34 | 84.29 | 59/70 |  | 94.55 | 94.82 | 476/502 |  |
| Wheezing | Yes | 49.57 | 51.43 | 36/70 |  | 0.00 | 0.00 | 0/502 | <0.001 |
|  | No | 50.43 | 48.57 | 34/70 |  | 100 | 100 | 502/502 |  |
| Hypertensive | Yes | 20.74 | 20.29 | 14/69 |  | 16.53 | 16.43 | 81/493 | 0.423 |
|  | No | 79.26 | 79.71 | 55/69 |  | 83.47 | 83.57 | 412/493 |  |
| **Rural** |  |  |  |  |  |  |  |  |  |
| **Males** |  |  |  |  |  |  |  |  |  |
| Cough | Yes | 63.61 | 67.05 | 59/88 |  | 21.26 | 19.20 | 198/1031 | <0.001 |
|  | No | 36.39 | 32.95 | 29/88 |  | 78.74 | 80.80 | 833/1031 |  |
| Shortness of breath | Yes | 42.85 | 43.18 | 38/88 |  | 5.72 | 5.14 | 53/1031 | <0.001 |
|  | No | 57.15 | 56.82 | 50/88 |  | 94.28 | 94.86 | 978/1031 |  |
| Chest pain | Yes | 59.46 | 61.36 | 54/88 |  | 21.86 | 20.93 | 216/1032 | <0.001 |
|  | No | 40.54 | 38.64 | 34/88 |  | 78.14 | 79.07 | 816/1032 |  |
| Sputum production | Yes | 39.32 | 39.77 | 35/88 |  | 5.25 | 5.04 | 52/1032 | <0.001 |
|  | No | 60.68 | 60.23 | 53/88 |  | 94.75 | 94.96 | 980/1032 |  |
| Wheezing | Yes | 72.39 | 75.00 | 66/88 |  | 0.00 | 0.00 | 0/1032 | <0.001 |
|  | No | 27.61 | 25.00 | 22/88 |  | 100 | 100 | 1032/1032 |  |
| Hypertensive | Yes | 11.89 | 12.64 | 11/87 |  | 6.61 | 7.40 | 76/1027 | 0.080 |
|  | No | 88.11 | 87.36 | 76/87 |  | 93.39 | 92.60 | 951/1027 |  |
| **Females** |  |  |  |  |  |  |  |  |  |
| Cough | Yes | 53.95 | 53.24 | 74/139 |  | 19.77 | 18.58 | 256/1378 | <0.001 |
|  | No | 46.05 | 46.76 | 65/139 |  | 80.23 | 81.42 | 1122/1378 |  |
| Shortness of breath | Yes | 39.79 | 39.57 | 55/139 |  | 6.25 | 6.46 | 89/1378 | <0.001 |
|  | No | 60.21 | 60.43 | 84/139 |  | 93.75 | 93.54 | 1289/1378 |  |
| Chest pain | Yes | 63.73 | 63.31 | 88/139 |  | 29.02 | 27.74 | 382/1377 | <0.001 |
|  | No | 36.27 | 36.69 | 51/139 |  | 79.98 | 72.26 | 995/1377 |  |
| Sputum production | Yes | 29.35 | 28.06 | 39/139 |  | 5.79 | 5.59 | 77/1378 | <0.001 |
|  | No | 70.65 | 71.94 | 100/139 |  | 94.21 | 94.41 | 1301/1378 |  |
| Wheezing | Yes | 79.01 | 79.86 | 111/139 |  | 0.00 | 0.00 | 0/1378 | <0.001 |
|  | No | 20.99 | 20.14 | 28/139 |  | 100 | 100 | 1378/1378 |  |
| Hypertensive | Yes | 23.80 | 22.63 | 31/137 |  | 14.63 | 14.60 | 200/1370 | 0.013 |
|  | No | 76.2 | 77.37 | 106/137 |  | 85.37 | 85.40 | 1170/1370 |  |

**Supplementary table 3. Factors associated with asthma (based on a multivariate model including only factors associated with asthma with p-value less than 0.05 at univariate analysis)**

| **Factors** | **With asthma**  **n (%)** | **Without**  **Asthma**  **n (%)** | **Crude estimates** | | **Adjusted estimates** | |
| --- | --- | --- | --- | --- | --- | --- |
|  |  |  | **Odds Ratio**  **(95% CI)** | **p-value** | **Odds Ratio (95% CI)** | **p-value** |
| **History of /passive smoking** | | | | | | |
| Yes | 46 (14.24) | 196 (6.34) | 2.80 (1.89 – 4.14) | <0.001 | 2.88 (1.78 – 4.67) | <0.001 |
| No | 277 (85.76) | 2896 (93.66) | 1 |  | 1 |  |
| **Family history of asthmaᶲ** | | | | | | |
| Yes | 87 (26.93) | 290 (9.39) | 3.57 (2.68 – 4.76) | <0.001 | 3.00 (2.05 – 4.40) | <0.001 |
| No | 236 (73.07) | 2800 (90.61) | 1 |  | 1 |  |
| **History of TB treatment** | | | | | | |
| Yes | 10 (3.10) | 40 (1.30) | 2.59 (1.19 – 5.62) | 0.016 | 0.98 (0.34 – 2.79) | 0.970 |
| No | 313 (96.90) | 3048 (98.70) | 1 |  | 1 |  |
| **Nasal congestion in the past 12 months** | | | | | | |
| Yes | 130 (40.25) | 408 (13.20) | 5.06 (3.79 – 6.75) | <0.001 | 3.29 (2.27 – 4.76) | <0.001 |
| No | 193 (59.75) | 2684 (86.80) | 1 |  | 1 |  |
| **Itchy-watery eyes in the past 12 months** | | | | | | |
| Yes | 131 (40.56) | 636 (20.56) | 2.78 (2.15 – 3.61) | <0.001 | 1.31 (0.93 – 1.84) | 0.118 |
| No | 192 (59.44) | 2457 (79.44) | 1 |  | 1 |  |
| **Skin rash in the past 12 months** | | | | | | |
| Yes | 67 (20.74) | 341 (11.04) | 2.16 (1.57 – 2.96) | <0.001 | 1.36 (0.90 – 2.06) | 0.142 |
| No | 256 (79.26) | 2748 (88.96) | 1 |  | 1 |  |
| **Cough** | | | | | | |
| Yes | 167 (51.70) | 544 (17.60) | 6.48 (4.76 – 8.82) | <0.001 | 2.39 (1.64 – 3.48) | <0.001 |
| No | 156 (48.30) | 2547 (82.40) | 1 |  | 1 |  |
| **Shortness of breath** | | | | | | |
| Yes | 130 (40.25) | 179 (5.79) | 14.24 (9.90 – 20.50) |  | 7.11 (4.71 – 10.74) | <0.001 |
| No | 193 (59.75) | 2911 (94.21) | 1 |  | 1 |  |
| **Chest pain** | | | | | | |
| Yes | 183 (56.66) | 690 (22.32) | 5.35 (4.04 – 7.08) | <0.001 | 2.91 (2.08 – 4.07) | <0.001 |
| No | 140 (43.34) | 2402 (77.68) | 1 |  | 1 |  |
| **Sputum production** | | | | | | |
| Yes | 92 (28.48) | 165 (5.33) | 9.01 (6.22 – 13.07) | <0.001 | 1.83 (1.15 – 2.91) | 0.010 |
| No | 231 (71.52) | 2928 (94.67) | 1 |  | 1 |  |
| **Exposure to bio-mass†** | | | | | | |
| Yes | 90 (27.95) | 608 (19.66) | 1.60 (1.20 – 2.14) | 0.001 | 1.28 (0.88 – 1.86) | 0.203 |
| No | 232 (72.05) | 2485 (80.34) | 1 |  | 1 |  |
| **Residence** | | | | | | |
| Urban | 96 (29.72) | 683 (22.08) | 1.48 (1.11 – 1.97) | 0.007 | 1.30 (0.84 – 2.00) | 0.243 |
| Rural | 227 (70.28) | 2410 (77.92) |  |  | 1 |  |
